# Drug-induced Angioedema: A Rare Side Effect of Rosuvastatin

**DOI:** 10.7759/cureus.2965

**Published:** 2018-07-11

**Authors:** Amir Shahbaz, Rupak Mahendhar, Mina Fransawy Alkomos, Paria Zarghamravanbakhsh, Issac Sachmechi

**Affiliations:** 1 Internal Medicine, Icahn School of Medicine at Mount Sinai/Queen Hospital Center, New York, USA; 2 Internal Medicine, Icahn School of Medicine, Mount Sinai/Queens Hospital Center, New York, USA; 3 Research, California Institute of Behavioral Neurosciences & Psychology, Sacramento, USA; 4 Endocrinology, Icahn School of Medicine at Mount Sinai Queen Hospital Center, New York, USA; 5 Internal Medicine, Icahn School of Medicine at Mount Sinai/Queens Hospital Center, New York, USA

**Keywords:** angioedema, rosuvastatin

## Abstract

Hydroxymethyl glutaryl coenzyme A reductase inhibitors (statins) are first-line medication for lowering serum cholesterol levels in the prevention of cardiovascular disease. Angioedema is the swelling of mucosa and submucosal tissue. There are no published cases of drug-induced angioedema involving rosuvastatin. We report a case of a 45-year-old female who presented with episodes of self-resolving edema of face, lips, and tongue after being on rosuvastatin. The patient denied any rash during these episodes and mentioned that self-medication with diphenhydramine did not relieve her symptoms. The patient was hemodynamically stable. The complement component 4 (C4), C1 esterase inhibitor, and complement component 1q (C1q) binding assay were within normal range. Therefore, the diagnosis of hereditary angioedema was effectively ruled out. The temporal relation between rosuvastatin and the development of angioedema and prompt resolution of symptoms after the drug discontinued suggest that rosuvastatin was the most probable culprit in the development of angioedema in our patient.

## Introduction

Hydroxymethyl glutaryl coenzyme A reductase inhibitors (statins) are first-line medication for lowering serum cholesterol in both primary and secondary prevention of cardiovascular disease. While statins are safe drugs in clinical practice, serious adverse effects such as elevated liver enzymes and myositis can happen [1]. Drug-induced non-inflammatory angioedema is a self-resolving but distressing side effect of some medications, e.g., angiotensin-converting enzyme inhibitors. There is an association between drug-induced angioedema and statin use in postmarketing reports [2]. There are no published cases of drug-induced angioedema involving rosuvastatin. We present a case of a 45-year-old female who presented with episodes of self-resolving edema of the face, lips, and tongue after being on rosuvastatin.

## Case presentation

A 45-year-old female patient with a past medical history of hypothyroidism and hyperlipidemia presented with recurrent night episodes of facial, lip, and tongue swelling. She did not have any rash during these episodes. The patient denied any allergic reaction in the past. She had not eaten anything unusual or traveled recently. There was no family history of allergic reaction or atopy. Self-medication with diphenhydramine did not relieve her symptoms. The patient was hemodynamically stable. Laboratory findings were not significant, and the eosinophilic count was normal. We reviewed her medications; she was taking levothyroxine 125 mcg daily for the last three years, and her thyroid function tests were stable. Two months back, rosuvastatin 20 mg was added for hyperlipidemia. Since that time, she had episodes of facial, lip, and tongue swelling that woke her up almost every night. The possible trigger of these episodes of angioedema was rosuvastatin, and we discontinued it. Complement component 4 (C4), C1 esterase inhibitor, and complement component 1q (C1q) binding assays were ordered. The patient's facial, lip, and tongue swelling resolved over the next 24 hours without the use of any further corticosteroid treatment. The patient's C4, C1 esterase inhibitors, and C1q binding assay were within normal range. Therefore, the diagnosis of hereditary angioedema (HAE) was ruled out. The temporal relation between rosuvastatin and the development of angioedema and prompt resolution of symptoms after drug discontinuation suggest that rosuvastatin was the most probable culprit for the development of angioedema in our patient. She was discharged home and colesevelam was started instead of rosuvastatin for hyperlipidemia. No other events were reported on follow-up visits and the patient was stable.

## Discussion

Angioedema is the swelling of mucosa and submucosal tissue and presents as the swelling of the face, lips, and tongue. It can be severe and life-threatening when it involves the respiratory tract. Medications that commonly cause drug-induced angioedema include angiotensin-converting enzyme inhibitor and non-steroidal anti-inflammatory drugs [3]. Allergic and non-allergic angioedema are two different types of drug-induced angioedema. Drug-induced allergic angioedema is a type I hypersensitivity reaction and mediated by histamine. It presents with a rapid onset of swelling of mucosa and submucosal tissues as well as a typical urticarial rash. Symptoms respond quickly to antihistamine, epinephrine, and corticosteroid treatment. Bradykinin is the primary mediator of drug-induced non-allergic angioedema. The onset is more progressive as compared to histamine-mediated angioedema. Symptoms may diminish in two to five days and are resistant to antihistamine and corticosteroid treatment. The discontinuation of the drug led to the resolution of drug-induced non-allergic angioedema [4].

Two mechanisms induce non-allergic angioedema in statin. Lovastatin has been shown to upregulate the expression of bradykinin type 2 receptors on endothelial cells in human coronary arteries. Statins may also increase the action of bradykinin on its receptors. Both mechanisms can make a patient susceptible to develop angioedema with circulating level of bradykinin through the increased release of prostacyclin and nitric oxide [5]. A detailed review of this patient's case, using the Naranjo probability scale, shows a probable association between rosuvastatin and angioedema [6]. Positively associated factors included previous postmarketing reports, occurrence after the administration of rosuvastatin, and improvement after stopping statin therapy. A review of the literature revealed case reports of statin-induced angioedema. These reports described a case of atorvastatin-induced angioedema with rash and eosinophilia, pravastatin-induced angioedema, lovastatin-induced angioedema with urticaria, and simvastatin-induced angioedema. Time of onset of these reactions varies from two days to nine months (Table 1) [2,7-9].

**Table 1:** List of reported cases of angioedema associated with statin use

Author	Age/Sex	Statin	Presentation
Nisly SA et al. [2]	75/F	Simvastatin	Recurrent face, lip, and tongue swelling
Hampson JP et al. [7]	67/M	Atorvastatin	Angioedema, hypotension
Liebhaber MI et al. [8]	54/M	Lovastatin	Urticaria, Angioedema
	49/M	Pravastatin	Urticaria, Angioedema
	77/F	Pravastatin	Urticaria, Angioedema
EI Mekki AB et al. [9]	43/F	Simvastatin	Swelling of face and lips

There are no laboratory-based tests available to provide immediate guidance on treatment. There is always a decrease in C4 level in hereditary angioedema. Tryptase is elevated in allergic angioedema. Discharge the patient after 12 to 24 hours of observation if there is no involvement of the tongue, larynx, or any other airway compromise. Standard treatment for histamine-mediated angioedema includes H1 and H2 antagonists, corticosteroids, and may require epinephrine in life-threatening situations. The only potential acute treatment currently readily available for the treatment of bradykinin-mediated angioedema in an emergency is fresh frozen plasma. Targeted therapies including icatibant, ecallantide, and C1-inhibitor concentrate are approved by the Food and Drug Administration (FDA) for the treatment of acute HAE attacks. They may have benefits in drug-induced nonallergic angioedema, but data are limited to support these treatments for non-HAE patients [10]. Adverse drug reaction will undoubtedly have a significant impact on the general management of a patient, as it will contraindicate not only that drug, but also those that are chemically related. Adverse drug reactions are rarely specific; diagnostic tests are usually absent, and a drug rechallenge is not ethically justified [11].

## Conclusions

We report a probable association between rosuvastatin and angioedema that improved significantly after cessation of medication. Physicians should consider this adverse effect in patients with persistent angioedema who are receiving rosuvastatin, or other statins.
